# Integrated multi-omic analysis identifies fatty acid binding protein 4 as a biomarker and therapeutic target of ischemia–reperfusion injury in steatotic liver transplantation

**DOI:** 10.1007/s00018-023-05110-1

**Published:** 2024-02-10

**Authors:** Mengfan Yang, Wenzhi Shu, Xiangyu Zhai, Xinyu Yang, Huaxin Zhou, Binhua Pan, Changbiao Li, Di Lu, Jinzhen Cai, Shusen Zheng, Bin Jin, Xuyong Wei, Xiao Xu

**Affiliations:** 1 Department of Organ Transplantation, Qilu Hospital of Shandong University, Jinan, 250012 China; 2 Zhejiang University School of Medicine, Hangzhou, 310058 China; 3 Key Laboratory of Integrated Oncology and Intelligent Medicine of Zhejiang Province, Hangzhou, 310006 China; 4 Department of Hepatobiliary Surgery, The Second Hospital, Shandong University, Jinan, 250033 China; 5 Department of Hepatobiliary and Pancreatic Surgery, Affiliated Hangzhou First People’s Hospital, Zhejiang University School of Medicine, Hangzhou, 310006 China; 6 Organ Transplantation Center, Affiliated Hospital of Qingdao University, Qingdao, 266035 China; 7 State Key Laboratory for Diagnosis and Treatment of Infectious Diseases, Hangzhou, 310003 China; 8 Department of Hepatobiliary and Pancreatic Surgery, The First Affiliated Hospital, Zhejiang University School of Medicine, Hangzhou, 310003 China

**Keywords:** Liver steatosis, Liver transplantation, Fatty acid binding protein 4, Mitochondrion, Proteomics, Transcriptomics, Metabolomics

## Abstract

**Background and aims:**

Due to a lack of donor grafts, steatotic livers are used more often for liver transplantation (LT). However, steatotic donor livers are more sensitive to ischemia–reperfusion (IR) injury and have a worse prognosis after LT. Efforts to optimize steatotic liver grafts by identifying injury targets and interventions have become a hot issue.

**Methods:**

Mouse LT models were established, and 4D label-free proteome sequencing was performed for four groups: normal control (NC) SHAM, high-fat (HF) SHAM, NC LT, and HF LT to screen molecular targets for aggravating liver injury in steatotic LT. Expression detection of molecular targets was performed based on liver specimens from 110 donors to verify its impact on the overall survival of recipients. Pharmacological intervention using small-molecule inhibitors on an injury-related target was used to evaluate the therapeutic effect. Transcriptomics and metabolomics were performed to explore the regulatory network and further integrated bioinformatics analysis and multiplex immunofluorescence were adopted to assess the regulation of pathways and organelles.

**Results:**

HF LT group represented worse liver function compared with NC LT group, including more apoptotic hepatocytes (*P* < 0.01) and higher serum transaminase (*P* < 0.05). Proteomic results revealed that the mitochondrial membrane, endocytosis, and oxidative phosphorylation pathways were upregulated in HF LT group. Fatty acid binding protein 4 (FABP4) was identified as a hypoxia-inducible protein (fold change > 2 and *P* < 0.05) that sensitized mice to IR injury in steatotic LT. The overall survival of recipients using liver grafts with high expression of FABP4 was significantly worse than low expression of FABP4 (68.5 vs. 87.3%, *P* < 0.05). Adoption of FABP4 inhibitor could protect the steatotic liver from IR injury during transplantation, including reducing hepatocyte apoptosis, reducing serum transaminase (*P* < 0.05), and alleviating oxidative stress damage (*P* < 0.01). According to integrated transcriptomics and metabolomics analysis, cAMP signaling pathway was enriched following FABP4 inhibitor use. The activation of cAMP signaling pathway was validated. Microscopy and immunofluorescence staining results suggested that FABP4 inhibitors could regulate mitochondrial membrane homeostasis in steatotic LT.

**Conclusions:**

FABP4 was identified as a hypoxia-inducible protein that sensitized steatotic liver grafts to IR injury. The FABP4 inhibitor, BMS-309403, could activate of cAMP signaling pathway thereby modulating mitochondrial membrane homeostasis, reducing oxidative stress injury in steatotic donors.

**Graphical Abstract:**

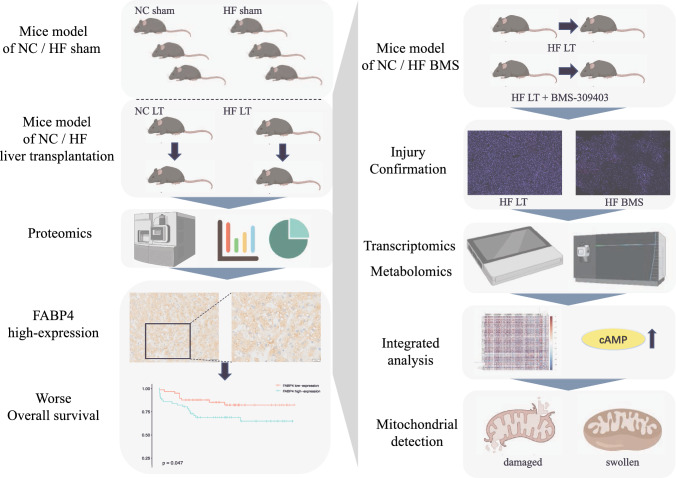

**Supplementary Information:**

The online version contains supplementary material available at 10.1007/s00018-023-05110-1.

## Introduction

Liver transplantation (LT) has become the most successful treatment for end-stage liver disease since the first LT procedure in the 1960s [1]. According to the statistics of the Chinese Liver Transplantation Registry (CLTR), from 2015 to 2020, the total cases of LT in China reached over 29,000, ranking second globally. The biggest issue for the LT community is the huge gap between donor graft demand and supply. Considering the ongoing level of obesity, diabetes, and metabolic syndrome, the prevalence of non-alcoholic fatty liver disease (NAFLD) is projected to continue to increase by over 25% globally [2, 3]. The increasing prevalence of NAFLD has reduced the donor pool, and steatotic grafts have been adopted more frequently.

The steatotic donor liver is more vulnerable to cold and warm ischemic injury than the normal liver, which worsens LT prognosis, including overall mortality and graft-specific morbidity and mortality [4, 5]. Efforts to optimize steatotic liver graft use have become a hot issue in clinical practice. Increasing proteomics researches revealed that proteins accompanied by changes in expression patterns could reveal the underlying target of liver injury [6]. Multi-omics integrative analysis is promising for understanding liver disease pathogenesis [7, 8]. Transcriptomics could detect the complete set of RNA transcripts, offer changes in transcript levels, and provide a better understanding of these differences. Metabolomics can offer a global metabolic profile and indicate metabolic disturbances, particularly in steatotic livers.

Consequently, high-fat diet and normal diet donor mouse LT models were established, and 4D label-free proteomics was adopted within the sham and LT groups to investigate the potential mechanisms and essential targets of ischemia–reperfusion (IR) injury in steatotic LT. Transcriptomics and metabolomics were used to investigate how small-molecule inhibitors protect steatotic donor LT from IR injury. These results would promote an understanding of heterogeneity between ideal and steatotic donor sources and provide a basis for targeted treatment of steatotic grafts for LT, thereby improving the prognosis of recipients and grafts.

## Materials and methods

### Animals

Six-week-old C57BL/6 mice were purchased from GemPharmatech Company (Nanjing, China). Two types of liver grafts were built through different diets from the age of eight weeks: donors in the fatty graft group (*n* = 6/group) were fed a high-fat diet (D12492, Research Diets) for eight weeks to produce moderately fatty liver, while donors in the normal graft group and all recipients were fed a normal diet. All mice were maintained in autocephalous individually ventilated cages (IVC) in a specific pathogen-free environment. All animal experiments followed the National Institute Guide for the Care and Use of Laboratory Animals. The Ethics Committee for the Use of Experimental Animals of Zhejiang University approved the experimental protocol.

### Establishment of mouse liver transplantation model

Mouse LT was performed in four groups: (i) sham operation in the normal control graft group (NC SHAM group), (ii) sham operation in the high-fat graft group (HF SHAM group), (iii) normal control donor liver transplantation group (NC LT group), and (iv) high-fat donor liver transplantation group (HF LT group). The other two groups of mouse LT were performed after confirmation of the injury target screened by proteomic analysis: (v) normal control donor liver transplantation group treated with fatty acid binding protein 4 (FABP4) inhibitor (BMS-309403, MedChemExpress) group (NC BMS group), and (vi) high-fat donor liver transplantation treated with FABP4 inhibitor group (HF BMS group). Each group contained six liver transplants. Liver tissue and blood samples were collected 6 h after surgery.

Two licensed animal surgeons performed mouse orthotropic LT as described in previous studies [9, 10]. FABP4 inhibitor, BMS-309403, was administered at 5 mg/kg body weight, and 120 µL of the BMS-309403 mixture was injected intraperitoneally into donor mice of the NC BMS and HF BMS groups 1 h before liver graft procurement. Then, LT was routinely performed.

Under inhalation anesthesia, donors were injected with heparin and cross-incised to expose the liver. The detached donor liver was perfused and immersed in a cold University of Wisconsin (UW) solution and then implanted orthotopically into the abdomen of the recipient mouse. The suprahepatic inferior vena cava was connected using a continuous suturing approach. The portal vein and the infra hepatic inferior vena cava were reconstructed by the “double cuff” technique. The common bile duct is associated with an indwelling biliary stent. The hepatic artery was not reconstructed. Standard rodent chow and sterilized water were available ad libitum after transplantation.

### Clinical sample preparation

Patients who underwent LT at the First Affiliated Hospital of Zhejiang University School of Medicine provided clinical samples between January 2016 and January 2017. In total, 110 samples of donor liver tissues were formalin-fixed paraffin-embedded (FFPE) and subjected to tissue microarray analysis. All recipients had individual hospital ID numbers, and their clinical characteristics were collected, including donor graft steatosis, post-LT liver function, the overall survival of recipients, and the survival of grafts. This study was approved by the Research Medical Ethical Committee of Zhejiang University School of Medicine’s First Affiliated Hospital’s regulations.

### Histological analysis

The liver tissue of mice was trimmed, embedded in optimal cutting temperature compound, and sliced into 5-μm-thick sections. Oil Red O (ORO) staining was used to assess the degree of liver steatosis. Two independent investigators blindly examined all tissue sections.

Hematoxylin–eosin (H&E) staining was performed on FFPE liver tissue and sectioned to a thickness of 5 μm. Then, the sections were stained with HE staining. The severity of liver injury was graded by Suzuki’s criteria.

Immunohistochemical (IHC) staining was performed using liver tissues from FFPE donors sectioned to a thickness of 4 μm. Briefly, 400 μL of diluted Anti-FABP4 primary antibody (ab92501; Abcam) was added to the slides. The histochemistry score was further utilized to transform the number of positive cells and staining intensity in each section into the corresponding values. The staining intensity scores were defined as 0 (no staining), 1 (weak positive), 2 (moderate positive), and 3 (strong positive). The positive areas were evaluated according to the percentage of positive cells: 0 (negative), 1 (1–25%), 2 (26–50%), 3 (51–75%), and 4 (76–100%). The final combined scores for each patient were decided, where the median score was regarded as the cutoff and divided into high- and low-expression groups.

### Terminal deoxynucleotidyl transferase-mediated dUTP nick end labeling (TUNEL) assay

DNA fragments from necrosis/apoptosis in mouse liver slides were detected using an in situ apoptosis detection kit (11684817; Roche). TUNEL assay was conducted following the manufacturer’s instructions.

### Serum transaminase assay

Serum ALT, AST, TB, and LDH levels were detected using the corresponding detection kits (C009-2-1, C010-2-1, C019-1-1, A020-2-2, Nanjing Jiancheng Bioengineering Institute). Serum transaminase levels were analyzed according to the manufacturer’s instructions.

### Western blot assay

Western blotting was performed to examine the expression of the specific target proteins. Total protein fractions from mouse liver were extracted, separated by gel electrophoresis, and transferred onto PVDF membranes (Millipore, MA, USA). Primary antibodies were incubated at 4 °C overnight and listed in Supplementary Table 1. Protein band densitometry was performed using Millipore ECL Western Blot Analysis Substrate (Merck Millipore, Germany).

### Proteomic analysis and database search

Mouse liver protein was extracted using tissue grinding and an ultrasonic processor. Trypsin was added for protein solution digestion. The tryptic peptides were dissolved in formic acid and acetonitrile/water and then directly loaded onto a reversed-phase analytical column. Peptides were separated to 80% and then held, all at a constant flow rate on a nanoElute UHPLC system (Bruker Daltonics). The peptides were subjected to a capillary source, followed by timsTOF Pro (Bruker Daltonics) mass spectrometry (MS). The resulting MS/MS data were processed using the MaxQuant search engine (v.1.6.15.0).

### Oxidative stress injury assay

Glutathione (GSH) in mouse liver tissue was detected using a GSH and oxidized glutathione disulfide (GSSG) Assay Kit (S0053, Beyotime). GSH content can be estimated from the sample control standard curve: GSH = Total GSH − GSSG × 2.

Malondialdehyde (MDA) in the mouse liver tissue was detected using the Lipid Peroxidation MDA Assay Kit (S0131S, Beyotime). The MDA concentration was estimated directly following the standard curve.

Superoxide dismutase (SOD) in the mouse liver tissue was detected using the Total SOD Assay Kit with NBT (S0109, Beyotime). The SOD activity was determined using the protein concentration and dilution ratio of the sample and control groups.

### Transcriptomic analysis and library preparation

Liver RNA was isolated. Poly-T oligo-attached magnetic beads enriched and purified mRNA from total RNA and broken it into 250–300 bp. First-strand cDNA was synthesized using random hexamer primers and Reverse Transcriptase. Second-strand cDNA synthesis was subsequently performed using the first strand of cDNA as a template. The library fragments were purified using the AMPure XP system (Beckman Coulter, Beverly, USA) to select cDNA fragments. Library quality was assessed using the Bioanalyzer 2100 system (Agilent Technologies, CA, USA). The index-coded samples were clustered using the cBot Cluster Generation System, and the library preparations were sequenced using next-generation sequencing (NGS) technology on the Illumina Novaseq platform (NEB, USA).

### Metabolomic and mass spectrometry analysis

Mouse liver tissues were quickly frozen and cut into Eppendorf tubes. Tissue samples and ceramic beads were then homogenized. For metabolite extraction, methanol and acetonitrile were added to the homogenized solution, and the supernatant was dried in a vacuum centrifuge after centrifugation. The analysis was performed using UHPLC (Vanquish UHPLC, Thermo) coupled to an Orbitrap. For HILIC separation, samples were analyzed using an ACQUIY UPLC BEH Amide 1.7 μm column (Waters, Ireland). The raw MS data were converted to MzXML files using ProteoWizard MSConvert, and the peaks were selected and grouped. Compound identification of metabolites was achieved by comparing the accuracy of m/z values (< 10 ppm) and MS/MS spectra with an in-house database established with available authentic standards.

### Bioinformatic methods

Gene ontology (GO) annotation divides proteins, genes, and metabolites into three categories: biological process, cellular compartment, and molecular function. The Kyoto encyclopedia of genes and genomes (KEGG) database was used to identify enriched pathways. A two-tailed Fisher’s exact test was used for each category to test the enrichment against all identified proteins, genes, and metabolites.

For hierarchical clustering based on functional classifications, we first compiled all categories acquired after enrichment alongside their P-values and then selected categories enriched in at least one of the clusters. Cluster membership was visualized with a heatmap using the “heatmap.2” function in the “gplots” R package.

All differentially expressed protein database accessions or sequences were searched against the STRING database for protein–protein interactions. Spearman correlation analysis determines the correlation coefficient between genes and metabolites with significant differences. The R package and Cytoscape were used to conduct matrix heatmaps, hierarchical clustering, and correlation network analysis to study the interaction between genes and metabolites from multiple perspectives.

### Reverse transcription-polymerase chain reaction (RT-PCR) assay

Total RNA was extracted from mouse livers using 1 mL of TRIzol reagent (Invitrogen, USA). RT-qPCR reactions were performed using the DNA Master SYBR Green Kit (Takara Bio, Japan) and Bio-Rad CFX96 Touch (Bio-Rad, Hercules, CA, USA) following the manufacturer’s instructions. Specific primers used for the target genes are listed in Supplementary Table 2. Relative mRNA quantification was performed using the 2^−∆∆Ct^ method, and all results were normalized to GAPDH mRNA expression.

### Transmission electron microscopy (TEM)

Mouse liver tissue was harvested at 1 mm^3^ and fixed using an electron microscope fixative. The tissue was then successfully subjected to fixation, dehydration, acetone penetration, embedding, sample sectioning at a thickness of 70 nm, and uranium–lead double staining. Mitochondria were detected using TEM (Hitachi, Tokyo, Japan).

### Multiplexed immunofluorescence staining

After deparaffinization, antigen retrieval, spontaneous fluorescence quenching, and bovine serum albumin blocking, FFPE slides of mouse livers were stained using multiplex immunofluorescence. Subsequently, the TUNEL reaction mixture and Rhodamine123 reagent were administered. The nuclei were stained with Hoechst after the antigens were labeled. Stained slides were scanned to obtain multispectral images using a confocal microscope (Olympus, Tokyo, Japan).

### Cell culture and treatment

The murine AML12 hepatocytes were routinely grown at 37 °C under 5% CO_2_ in DMEM-F12 cell culture media and 10% fetal bovine serum, without antimicrobial compounds. AML12 hepatocytes were stimulated with palmitic acid (PA) and oleic acid (OA) for 48 h to build steatotic hepatocyte [11]. FABP4 Small interfering RNA (siRNA) was purchased from Sangon Biotech (Shanghai) and transfected to decrease FABP4 level in AML12 cells.

Four groups of in vitro model were established, including normal control (NC) group, normal cells with FABP4 siRNA (NC + siRNA) group, high-fat (HF) group, and high-fat cells with FABP4 siRNA (HF + siRNA) group. Then the four groups of AML12 cells were subjected to 12 h of hypoxia, followed by 4 h of reoxygenation [12].

### Flow cytometry assay

The level of apoptosis was quantified by Annexin V–propidium iodide (PI) apoptosis detection kit (BD Biosciences, CA, United States). Firstly, AML-12 cells were digested and washed with pre-chilled PBS three times. Then cells were resuspended in binding buffer. Next, adding PI and Annexin V to stain apoptosis cells in turns. BD LSR flow cytometry was used to detect apoptosis and the data were analyzed by FlowJo.

### Statistical analysis

SPSS software (version 23.0; SPSS, Chicago, USA), R studio software (version 4.0.3), and Prism software (version 9.1.1, GraphPad Software, LLC, USA) were employed for data analysis. Western blot bands were examined using ImageJ software (Version 2.1.0, NIH, USA). Statistical tests used in the study included Student’s t test, Wilcoxon rank sum test, chi‐square test, two-tailed Fisher’s exact test, and Log-rank test. Measurement data are presented as the mean ± standard deviation, and a *t*-test was used for inter-group comparisons. For GO annotation and KEGG database analysis, a two-tailed Fisher’s exact test was employed to test the enrichment. STRING defines a metric called “confidence score” to define interaction confidence; we fetched all interactions that had a confidence score ≥ 0.7. The Wilcoxon rank sum test was applied to compare FABP4 expression levels of liver tissues, while the Pearson chi‐square test was used to compare categorical variables. Two-tailed Wilcoxon rank sum tests were used to compare mRNA and protein expression levels. The postoperative overall survival rates were examined using Kaplan–Meier curves, and the log-rank test was used for comparisons between the high- and low-expression groups. All statistical tests were bilateral, and significance was set at *P* < 0.05.

## Results

### Establishment and validation of mouse liver transplantation model

**Fig. 1:**
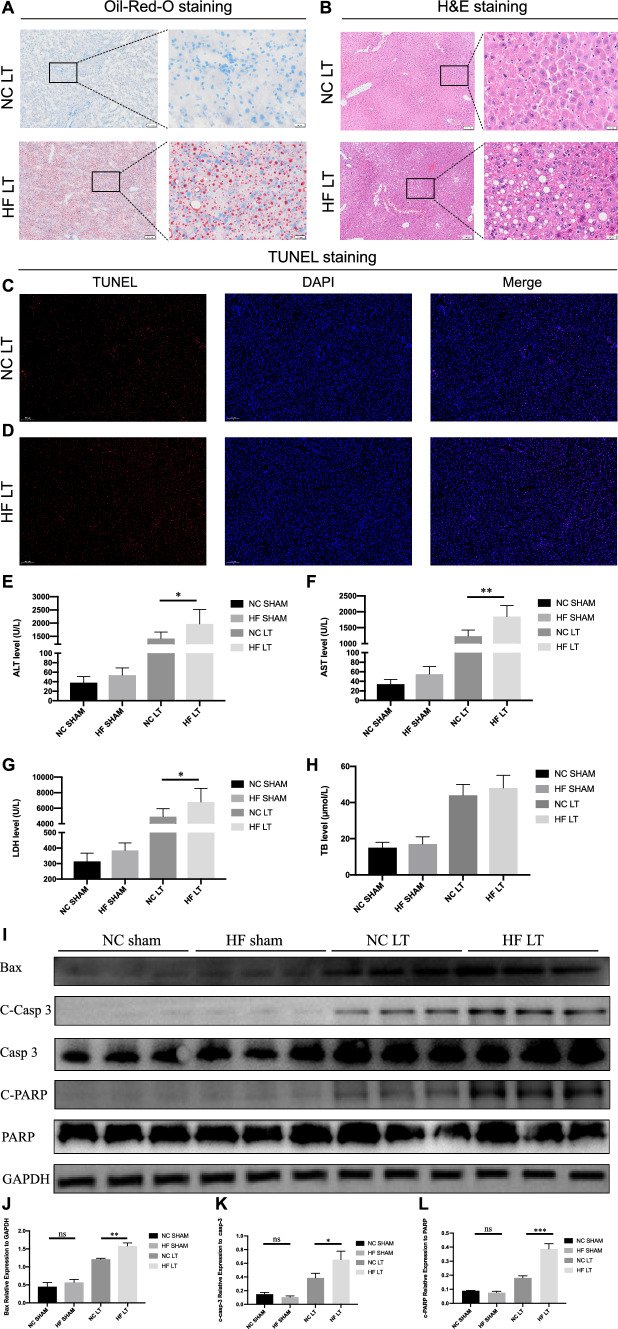
Establishment and validation of mouse liver transplantation model. **A** Oil Red O staining of mouse liver transplantation model. **B** H&E staining. **C**, **D** TUNEL staining. **E**–**H** Liver function detection of ALT, AST, LDH, and TB. **I**–**L** Relative expression of Bax, cleaved Caspase-3, and cleaved PARP. ns, not significant; *P < 0.05; **P < 0.01; ***P < 0.001; ****P < 0.001

Donor mice were fed different diets for eight weeks and transplanted into normal mice. Oil Red O staining was used to identify the success of fatty liver development (Fig. 1A). Hepatic steatosis in the HF LT group was 30–60% under the microscope, and no hepatic steatosis was observed in the NC LT group. H&E staining displayed considerable inflammatory cell infiltration and fat vacuoles in the HF LT group (Fig. 1B). TUNEL staining was performed, and more apoptotic liver cells were found in the HF LT group than in the NC LT group (Fig. 1C and D).

Liver function analysis demonstrated that serum ALT, AST, and LDH levels were significantly higher in the HF LT than in the NC LT group (1970 ± 550 vs. 1420 ± 221 U/L, *P* < 0.05; 1849 ± 349 vs. 1235 ± 194 U/L, *P* < 0.01; 6796 ± 1739 vs. 4918 ± 1029 U/L, *P* < 0.05; Fig. 1E–G). The relative expression of Bax, cleaved Caspase-3, and cleaved PARP was significantly higher in the HF LT than in the NC LT group (*P* < 0.01,* P* < 0.05,* P* < 0.001, respectively; Fig. 1J–L). All above results demonstrated severe hepatocellular damage in the HF LT group.

The corresponding NC and HF SHAM groups detection was conducted, including Oil Red O, H&E, and TUNEL staining (Supplementary Fig. 1).

### Proteomic profile revealing the change of FABP4 during liver transplantation

**Fig. 2:**
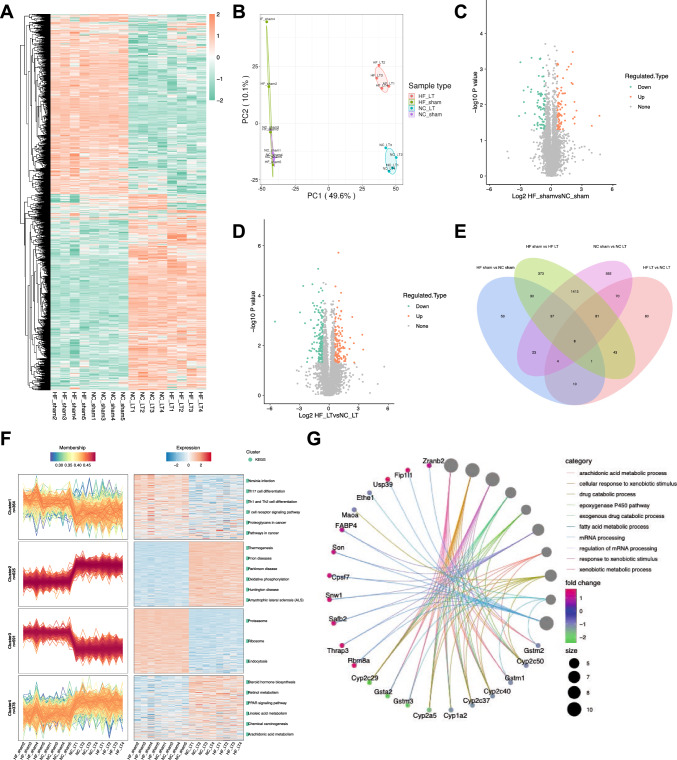
Proteomic profile revealing the change during liver transplantation. **A** Heatmap of the differentially expressed proteins. Red rectangles mean that proteins are upregulated, and green ones mean that they are downregulated. **B** Principal component analysis of the duplicate samples, in which the degree of aggregation among samples represents statistical consistency. **C** Volcano plot of the differentially expressed proteins between NC SHAM and HF SHAM groups. Gray dots represent genes that are not differentially expressed; red dots and blue dots represent genes that are upregulated and downregulated significantly. **D** Volcano plot of the differentially expressed proteins between NC LT and HF LT groups. **E** Venn diagram demonstrating shared and discrete proteins in each of these four groups. **F** Expression patterns of these differentially expressed proteins based on membership and expression. **G** Protein–protein interaction network of differentially expressed proteins with FABP4

Proteomics was adopted among the four groups, including NC SHAM, HF SHAM, NC LT, and HF LT. A total of 5131 quantifiable proteins were identified, and the differentially expressed proteins were revealed in a heatmap (Fig. 2A). Principal component analysis (PCA) depicted that duplicate samples were statistically consistent. The clustering degree between samples was quite dense, and circles representing the NC LT and HF LT groups were far from the SHAM circles and each other (Fig. 2B). The fold change and *P*-values were considered while screening for differentially expressed proteins. After screening, 168 proteins were identified between NC SHAM and HF SHAM groups (Fig. 2C), 297 proteins were identified between NC LT and HF LT groups (Fig. 2D), 2,241 proteins were identified between NC SHAM and NC LT groups, and 1994 proteins were identified between HF SHAM and HF LT groups. Shared and discrete proteins were identified among these groups (Fig. 2E).

The molecular function of the GO pathway indicated that fatty acid elongase activity, fatty acid synthase activity, and oxidoreductase activity were separated by enrichment analysis between NC SHAM and HF SHAM groups. In contrast, the KEGG pathway analysis revealed that ferroptosis and fatty acid elongation were screened. Compared with NC LT and HF LT groups, cellular components revealed that the mitochondrial protein complex, mitochondrial inner membrane, inner mitochondrial membrane protein complex, and endoplasmic reticulum were screened. KEGG pathway analysis revealed that the inflammatory mediator regulation of TRP channels was screened. Comparing HF SHAM and HF LT groups, biological processes found that the apoptotic signaling pathway, extrinsic apoptotic signaling pathway, and mitochondrial transmembrane transport were analyzed. The cellular components revealed that ribosomes, mitochondrial respirasomes, respiratory chain complexes, and mitochondrial protein complexes were screened. KEGG pathway analysis revealed autophagy and apoptosis in both groups. Clustering was used to assess the expression patterns of differentially expressed proteins based on their membership and expression (Fig. 2F). Clustering analysis of the four groups revealed significant changes in the expression profiles of the ribosomes, proteasomes, endocytosis, and oxidative phosphorylation pathways (Supplementary Fig. 2).

After analyzing the differently expressed proteins among all comparison groups, fatty acid binding protein 4 (FABP4), which also interacts with fatty acid metabolism and synthesis, was screened for changes in each pair of these four groups. The differential protein–protein interaction relationship was obtained using the String database, and the protein–protein interaction network was visually displayed (Fig. 2G).

### Relationship with FABP4 expression and liver transplantation recipients’ overall survival

**Fig. 3:**
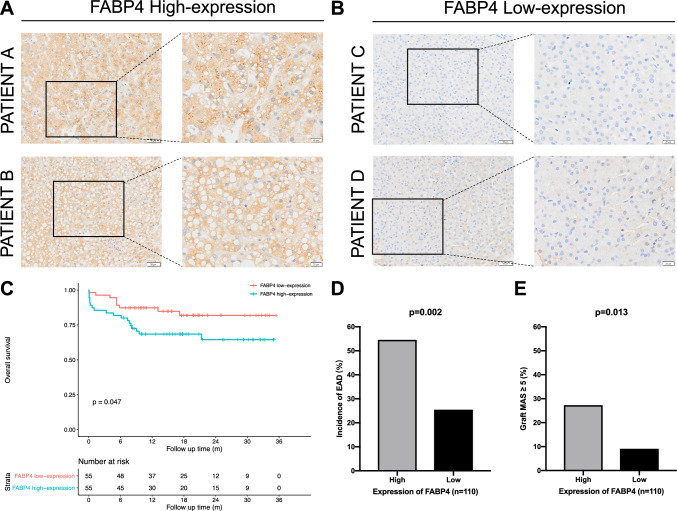
Relationship with FABP4 expression and liver transplantation recipients’ overall survival. **A** FABP4 is a high expression of donors’ liver tissue microarray IHC staining. **B** FABP4 low expression of IHC staining. **C** Comparison of overall survival rate between FABP4 high- and low-expression groups. **D** Comparison of the incidence of early allograft dysfunction. **E** Comparison of the risk of liver steatosis

A total of 110 LT liver tissue microarrays of donors were sectioned, and FABP4 IHC staining was performed. The clinical characteristics of donors and recipients, including prognostic follow-up, were collected. The histochemistry score was calculated for each sample. FABP4’s high expression (Fig. 3A) and low expression (Fig. 3B) were determined based on the median histochemistry score. A total of 55 FABP4 high- and 55 FABP4 low-expression cases were enrolled.

A survival curve was plotted based on FABP4 expression. The 1-year overall survival rate of the FABP4 high-expression group was 68.5%, which was significantly worse than that of the FABP4 low-expression group (68.5 vs. 87.3%, *P* < 0.05, Fig. 3C). The incidence of early allograft dysfunction (EAD) was relatively higher in recipients in the FABP4 high-expression group (54.5 vs. 25.5%, *P* < 0.01, Fig. 3D). In addition, donor grafts in the FABP4 high-expression group were often accompanied by liver steatosis (27.3 vs. 9.1%, *P* < 0.05, Fig. 3E).

### Influence of FABP4 inhibitor on mouse high fatty liver transplantation model

**Fig. 4:**
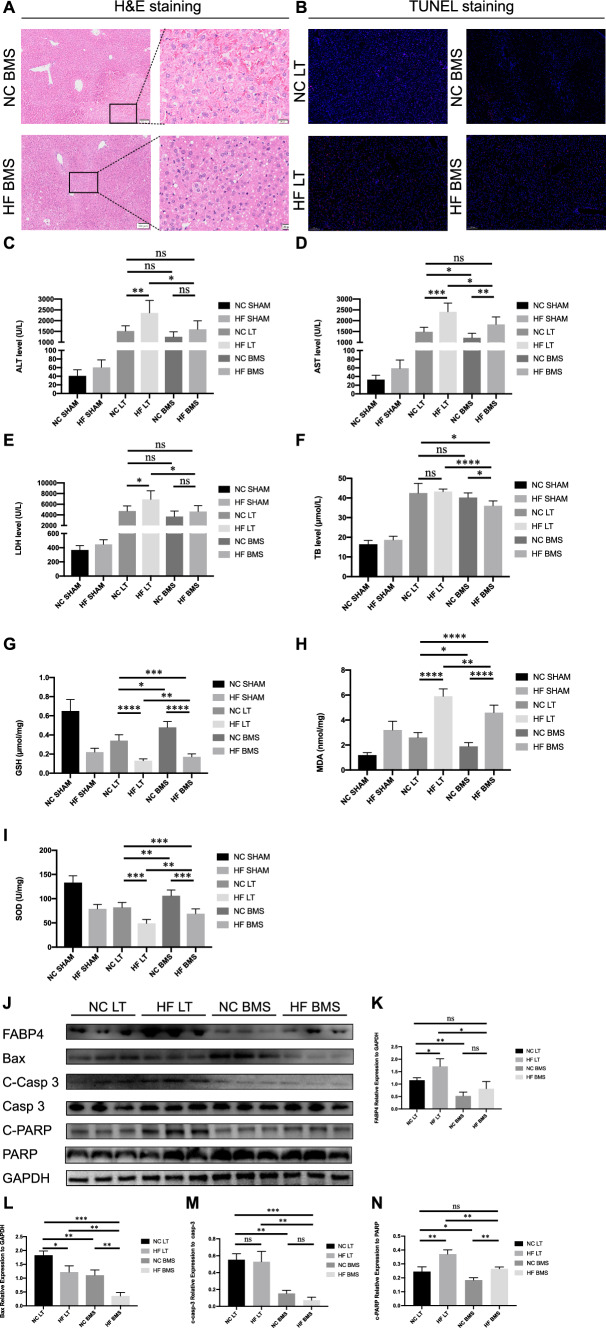
Influence of FABP4 inhibitor on mouse high fatty liver transplantation model. **A** H&E staining of mouse liver transplantation model after using FABP4 inhibitor. **B** TUNEL staining. **C**–**F** Liver function detection of ALT, AST, LDH, and TB. **G**–**I** Oxidative stress injury assay of GSH, MDA, and SOD. **J**–**N** Relative expression of FABP4, Bax, cleaved Caspase-3, and cleaved PARP. ns, not significant; *P < 0.05; **P < 0.01; ***P < 0.001; ****P < 0.001

One hour before donor liver procurement, the FABP4 inhibitor BMS-309403 was administered. H&E staining displayed that the liver had many fat vacuoles, a moderate decrease in inflammatory cell infiltration, and a considerable decrease in apoptotic hepatocytes in HF BMS compared with HF LT (Fig. 4A). A small amount of hepatocyte rupture and nuclear lysis was observed in the NC BMS group than in the NC LT group. TUNEL staining revealed a significant decrease in the number of apoptotic hepatocytes after BMS-309403 treatment (Fig. 4B).

Liver function detection revealed that serum ALT, AST, and LDH were significantly higher in the HF LT group than in the NC LT group (*P* < 0.01,* P* < 0.001,* P* < 0.05, respectively; Fig. 4C–E). These three indicators and TB decreased significantly after the adoption of BMS-309403 compared to the HF BMS and HF LT groups (*P* < 0.05,* P* < 0.05,* P* < 0.05, and *P* < 0.0001). However, this trend was not significant compared with NC BMS and NC LT groups (*P* > 0.05,* P* < 0.05,* P* > 0.05, and *P* > 0.05). The adoption of BMS-309403 could improve the liver function indicators in the HF BMS group compared to those in the NC LT group (*P* > 0.05,* P* > 0.05,* P* > 0.05,* P* < 0.05).

After BMS-309403 was used, the oxidative stress damage tests demonstrated substantial variations. The values of GSH and SOD decreased, and MDA levels increased in the HF BMS group compared with those in the HF LT group (*P* < 0.01,* P* < 0.01,* P* < 0.01, respectively; Fig. 4G–I). This trend was also observed when comparing NC BMS and NC LT groups (*P* < 0.05,* P* < 0.01, and* P* < 0.05). The relative expression of FABP4, Bax, cleaved Caspase-3, and cleaved PARP has decreased significantly in the HF BMS group compared to the HF LT group (*P* < 0.05,* P* < 0.01,* P* < 0.01, and* P* < 0.01, Fig. 4K–N). Correspondingly, the relative expression of these proteins was also lower in the NC BMS group than in the NC LT group (*P* < 0.01,* P* < 0.01,* P* < 0.01, and *P* < 0.05).

### Transcriptomic profiles revealing the influence of FABP4 inhibitor

**Fig. 5:**
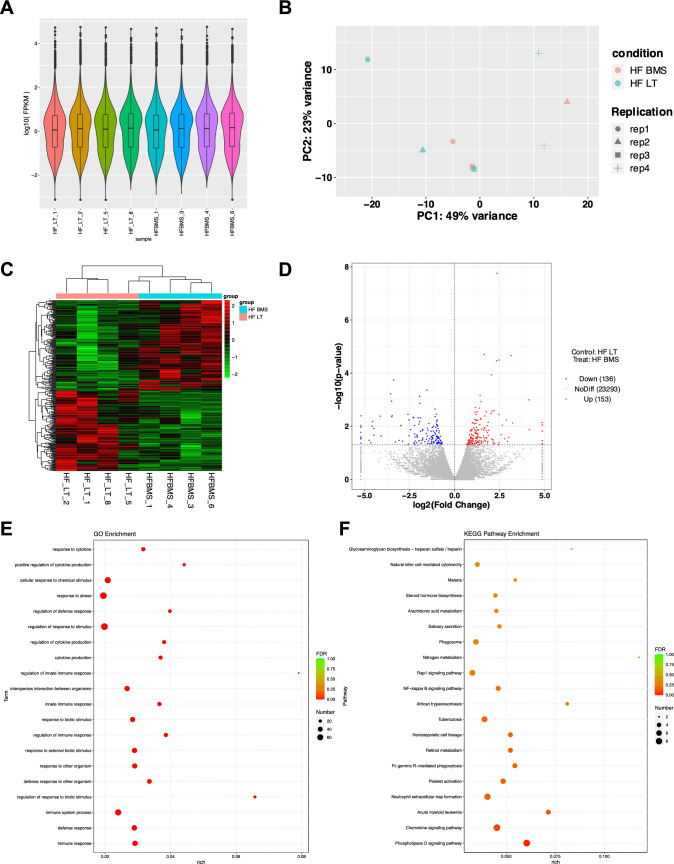
Transcriptomic profiles revealing the influence of FABP4 inhibitor. **A** Violin diagram of expression patterns of all genes. **B** Principal component analysis of the duplicate samples. **C** Heatmap of the differentially expressed genes. **D** Volcano plot of the differentially expressed genes between HF LT and HF BMS groups. **E** Gene ontology pathway enrichment for differentially expressed proteins. The circle sizes represent the number of genes enriched in pathways, and the circle’s color means significance. **F** KEGG pathway enrichment for differentially expressed proteins

Transcriptomics was used in HF LT and HF BMS groups. As displayed in Fig. 5A, all genes were moderately expressed. PCA revealed that duplicate samples conformed to statistical consistency (Fig. 5B). In total, 289 quantifiable genes were identified. The differentially expressed genes are shown in a heatmap (Fig. 5C), where 153 upregulated and 136 downregulated genes were identified and are depicted in a volcano map (Fig. 5D).

Immune response, defensive response, and stimulus regulation were screened by enrichment analysis of biological processes (Fig. 5E). Cellular components such as phagocytic vesicles, early phagosomes, and endoplasmic reticulum membranes were screened. Molecular function analysis revealed that ion binding and cation binding were screened. KEGG pathway analysis revealed Fc γ R-mediated phagocytosis, and phagosomes were screened (Fig. 5F). The pathway enrichment analysis is shown in Supplementary Fig. 3.

### Metabolomic profiles revealing the influence of FABP4 inhibitor

**Fig. 6:**
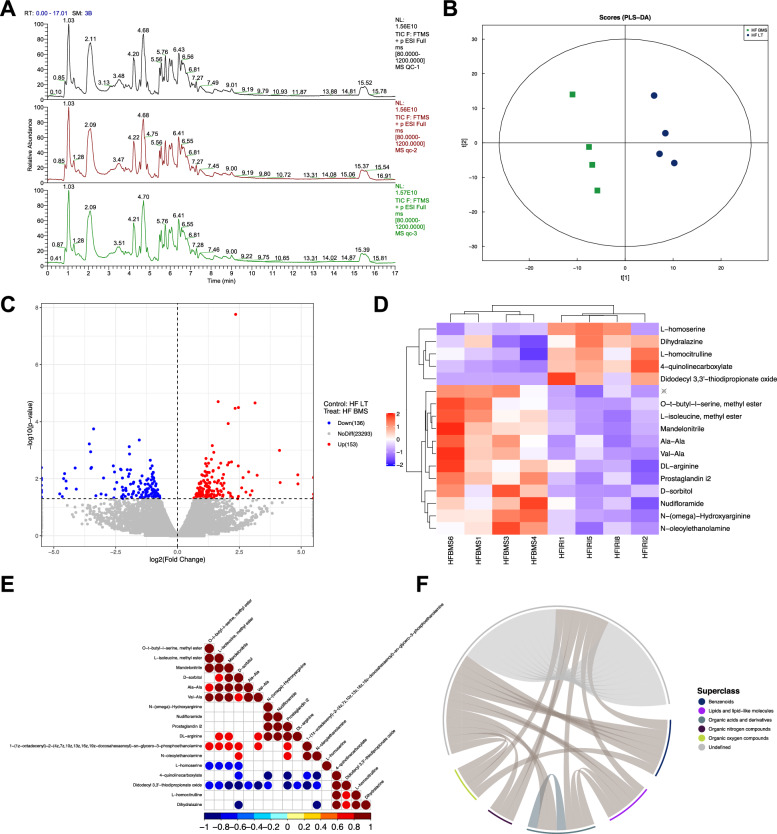
Metabolomic profiles revealing the influence of FABP4 inhibitor. **A** Total ion chromatogram of spectral overlap comparison, with response intensity and retention time overlapping. **B** Principal component analysis of the duplicate samples. **C** Volcano plot of the differentially expressed metabolites between HF LT and HF BMS groups. **D** Heatmap of the differentially expressed metabolites. **E** Correlation analysis between metabolites and visualized in the form of correlation heatmaps. **F** Revealing the co-regulatory relationships between various metabolites by chord diagrams

Metabolomics was also adopted to compare HF LT and HF BMS groups. The total ion chromatogram spectral overlap comparison displayed overlapping response intensities and retention times (Fig. 6A). PCA revealed that close clustering of the quality control samples indicated good repeatability of the experiment (Fig. 6B). A total of 159 upregulated and 105 downregulated metabolites were identified, as demonstrated in the volcano map (Fig. 6C). Cluster analysis of the differential metabolites demonstrated similar expression patterns for metabolites in the same cluster (Fig. 6D).

Metabolic proximities were analyzed to further understand the mutual regulatory relationships between metabolites during the biological state (Fig. 6E). A chord diagram of metabolic proximities also revealed a correlation between the various metabolites (Fig. 6F). The lipids and lipid-like compounds were tested for organic acids and their derivatives. Only the VEGF and cAMP signaling pathways were evaluated between the two groups by the KEGG pathway.

### Integrated transcriptomic and metabolomic analysis

**Fig. 7:**
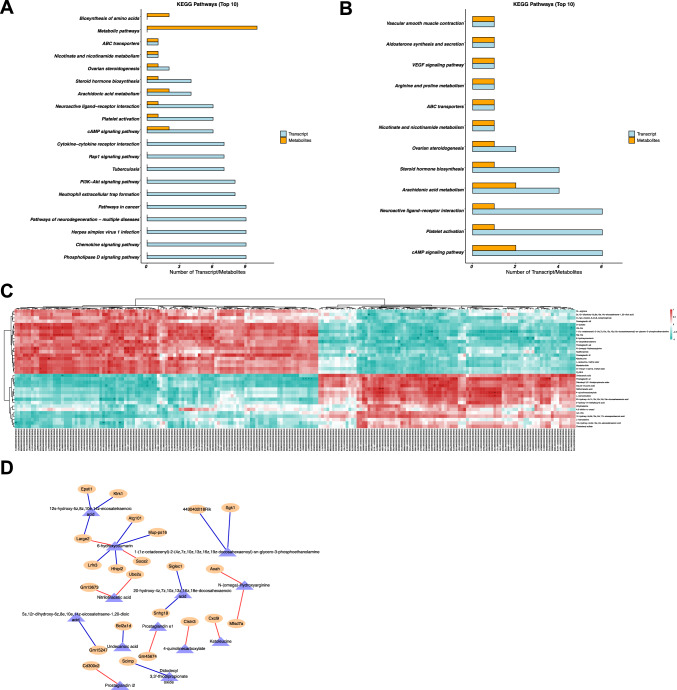
Integrated transcriptomic and metabolomic analysis. **A** KEGG pathways that transcriptomic and metabolomic respectively enriched. **B** KEGG pathways that transcriptomic and metabolomic simultaneously enriched. **C** Spearman’s correlation hierarchical clustering analysis of differences in the expression patterns. **D** Correlation network analysis of significant differences in key node locations

Visualizing gene and metabolite expression is important for interpreting high-throughput omics experiments to understand how IR injury in steatotic LT is initiated. Gene–metabolite interaction analysis was used to study and depict differential gene–metabolite interactions. Transcriptomics screened 216 pathways, and metabolomics screened 24 pathways together (Fig. 7A); 12 pathways were found to be involved in both genomes (Fig. 7B). Aldosterone synthesis and secretion, steroid hormone biosynthesis, and cAMP signaling pathways have more molecules annotated in the two groups for the biological pathway.

Spearman correlation hierarchical clustering was conducted on genes and metabolites with substantial differences to intuitively represent expression patterns (Fig. 7C). Different genes or metabolites that appear in the same cluster have similar expression patterns. Correlation network analysis was performed to screen for genes and metabolites with significant differences in key node locations (Fig. 7D). The metabolite 6-hydroxycoumarin was associated with six genes, two positively and four negatively correlated.

### Influence of FABP4 inhibitor on cAMP signaling pathway

**Fig. 8:**
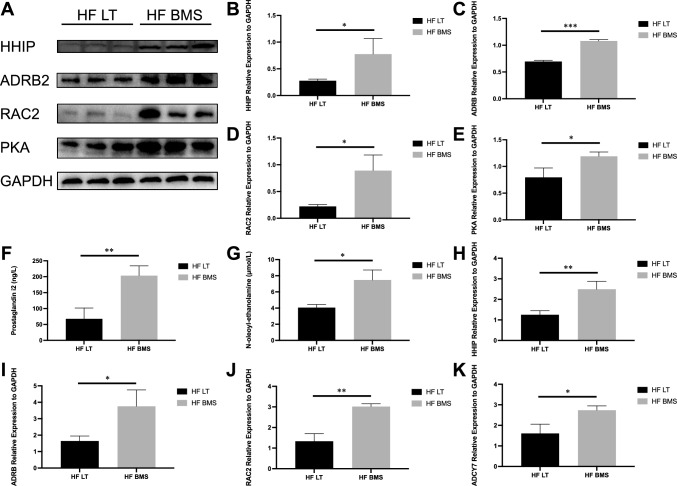
Influence of FABP4 inhibitor on cAMP signaling pathway. **A**–**E** Relative expression of HHIP, ADRB2, RAC2, and PKA. **F**, **G** The content of Prostaglandin i2 and N-oleoylethanolamine validated by ELISA. **H**–**K** The mRNA expression of Hhip, Adrb2, Rac2, and Adcy7 of HF LT and HF BMS groups. ns, not significant; *P < 0.05; **P < 0.01; ***P < 0.001; ****P < 0.001

Transcriptomic profiles revealed that 6 genes were differently expressed in cAMP signaling pathway, including 5 upregulated (Hhip, Adrb2, Rac2, Adcy7, and Rapgef4) and 1 downregulated (Grin3b). The metabolomic profiles revealed 2 upregulated metabolites (Prostaglandin i2 and N-oleoylethanolamine). Western blot assay revealed that the relative expression of Hhip, Adrb2, Rac2, and protein kinase A (PKA) was significantly higher in the HF BMS than in the HF LT group (*P* < 0.05, *P* < 0.001, *P* < 0.05, *P* < 0.05, respectively; Fig. 8B–E). The content of metabolites of two groups was also validated by ELISA. Prostaglandin i2 and N-oleoylethanolamine increased more than twofold after FABP4 inhibitors treatment (*P* < 0.01, *P* < 0.05; Fig. 8F, G). Moreover, the mRNA expression of Hhip, Adrb2, Rac2, and Adcy7 was elevated in HF BMS group, indicating activation of the cAMP signaling pathway (*P* < 0.01, *P* < 0.05, *P* < 0.01, *P* < 0.05, respectively; Fig. 8H–K). The adoption of FABP4 inhibitors might activate the cAMP signaling pathway.

### Influence of FABP4 inhibitor on the high fatty liver mitochondrion

TEM was used to observe the mitochondrial morphology of the mouse liver. There was no edema in the hepatocytes, and intracellular organelles were abundant in the HF SHAM group (Fig. 8A). The bile duct structure was in good condition. The nucleus was round, and the nuclear membrane was complete. Mitochondria are ovoid, shallow, and without bulging, fractured, or shortened cristae. Lipid droplets were scattered throughout the distribution. There were more autophagosomes and autolysosomes in the HF LT group (Fig. 8B), mitochondrial swelling, unclear structure, and partial disintegration of the ridge. The HF BMS group had an obvious mitochondrial structure, and the injured and healthy mitochondria were lytic, suggesting autophagy (Fig. 8C).

Mitochondrial membrane potential was detected using multiplex immunofluorescence staining (Fig. 8D–F). The staining results displayed a larger area of mitochondrial membrane potential loss in the HF LT group than in the HF SHAM group regarding mitochondrial membrane homeostasis. Membrane depolarization still existed but depicted a significant easing trend after adopting BMS-309403 in the HF BMS group. Compared to the HF LT group, the TUNEL fluorescence intensity in the HF BMS group was also weakened.

**Fig. 9:**
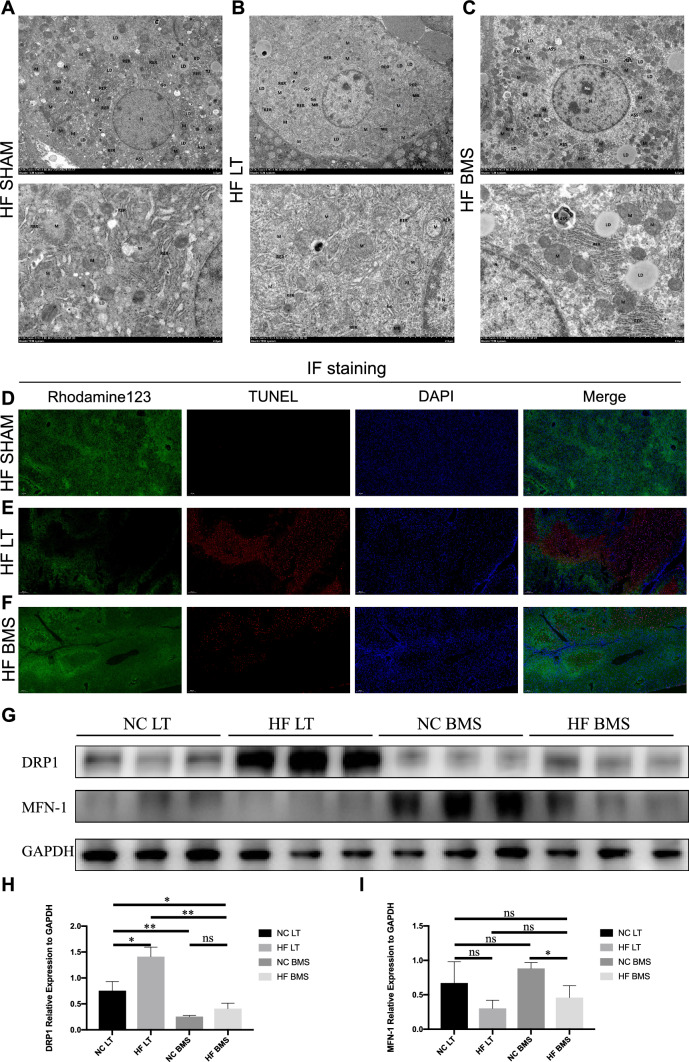
Influence of FABP4 inhibitor on high fatty liver mitochondrion. **A**–**C** Transmission electron microscopy image of mouse liver in each group. **A** The mitochondrial structure was clear, and no autophagosomes were presented in the HF SHAM group. **B** Autophagosomes and autolysosomes were found in the HF LT group with mitochondrial swelling. **C** The mitochondrial structure of the HF BMS group was clear, and damaged parts of mitochondria were lytic. **D**–**F** Multiplex immunofluorescence staining of rhodamine reagent for detecting mitochondrial membrane potential. Green fluorescence represents mitochondrial membranous potential, red fluorescence represents apoptotic hepatocytes, and blue fluorescence represents nuclear staining. **G**–**I** Relative expression of DRP1 and MFN-1. ns, not significant; *P < 0.05; **P < 0.01; ***P < 0.001; ****P < 0.001. AP, autophagosomes; ASS, autolysosome; LD, lipid droplets; M, mitochondria; RER, rough endoplasmic reticulum

Compared to HF LT, DRP1 expression decreased considerably in HF BMS (*P* < 0.01, Fig. 8H). The relative expression of MFN-1 in the HF BMS group displayed an increasing trend but was insignificant (Fig. 9).

### Influence of FABP4 siRNA on in vitro hypoxia / reoxygenation model

**Fig. 10:**
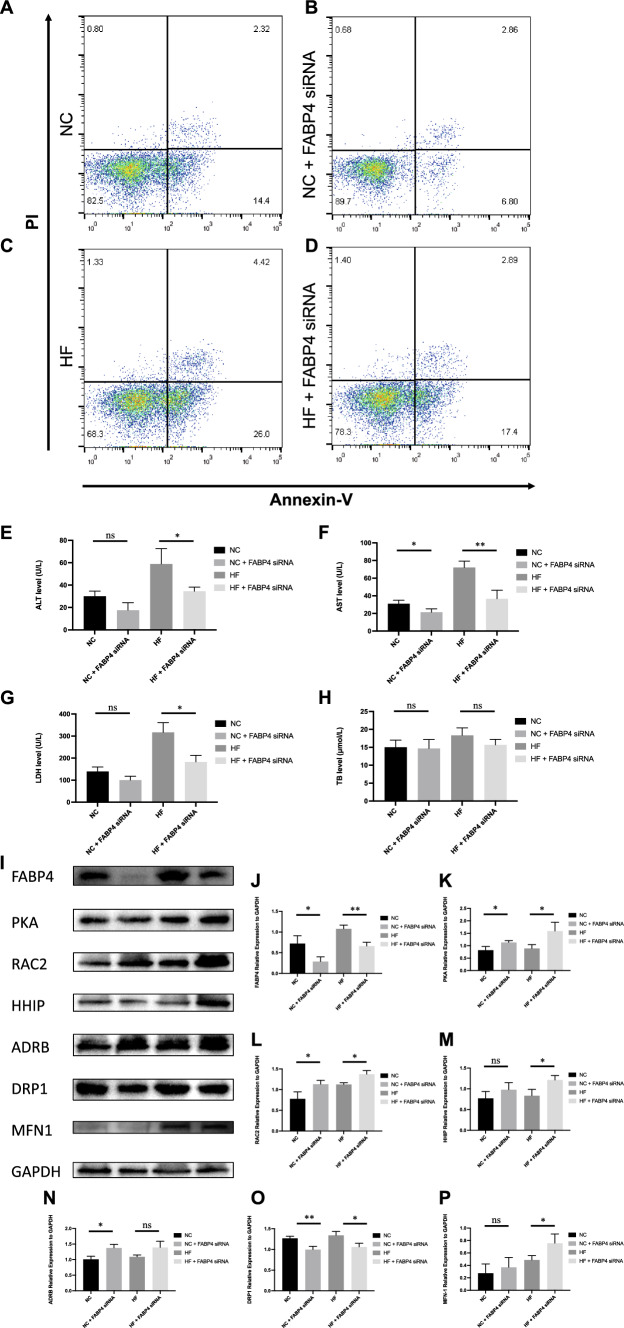
Influence of FABP4 siRNA on in vitro hypoxia / reoxygenation model. **A**–**D** Flow cytometry was performed to detect the hypoxic injury-induced apoptosis of AML12 cells. **E**–**H** Liver function detection of ALT, AST, LDH, and TB using the supernatant of each group. **I**–**P** Relative expression of FABP4, PKA, RAC2, HHIP, ADRB, DRP1, and MFN-1. ns, not significant; *P < 0.05; **P < 0.01; ***P < 0.001; ****P < 0.001

Flow cytometry was performed to detect the hypoxic injury-induced apoptosis of AML12 cells after the cells were stained with Annexin V / propidium iodide. As shown in Fig. 10, the apoptosis rate from the HF group (Fig. 10C) increased significantly compared with that of the NC group (Fig. 10A), while the HF siRNA group (Fig. 10D) had a lower apoptosis rate than the HF group. The supernatant of each group was collected for detecting ALT, AST, LDH, and TB. The levels of ALT, AST, and LDH were significantly higher in the HF group than in the NC group, which were significantly increased in HF siRNA group (*P* < 0.05, *P* < 0.01, *P* < 0.05, respectively; Fig. 4E–G). Thus, adoption of FABP4 siRNA in steatotic murine AML12 hepatocytes could protect from the hypoxia/reoxygenation injury.

Western blot assays were applied to demonstrate the activation of cAMP signaling pathway. The relative expression of PKA, Rac2, and Hhip was significantly higher in the HF siRNA group than in the HF group (*P* < 0.05, *P* < 0.05, *P* < 0.05, respectively; Response Fig. 4K–M). The relative expression of Adrb2 was also higher but not significant in the HF siRNA group than in the HF group (*P* > 0.05, Fig. 4N). Compared to HF group, DRP1 expression decreased considerably in HF siRNA group (*P* < 0.05, Response Fig. 4O). The relative expression of MFN-1 in the HF siRNA group displayed an increasing trend (*P* < 0.05, Fig. 4P), indicating the modulating mitochondrial membrane homeostasis role of FABP4 siRNA, which probably owing to the activation of cAMP signaling pathway.

## Discussion

NAFLD and alcoholic fatty liver disease (AFLD) have rapidly developed in China due to lifestyle changes [13, 14]. LT is an effective treatment for end-stage liver disease, but the shortage of donor livers has always been a bottleneck. The development and promotion of organ donation following citizen death have increased donor liver supplies, and marginal donor livers, including steatosis, have become a research hotspot [15, 16]. The “Technical Specifications for Obtaining Donor Liver for Liver Transplantation in China (2019 edition)” suggested that donor liver with micro-steatosis and mild macro-steatosis can be used routinely, and donor liver with moderate macro-steatosis should be evaluated carefully [17]. Studies have shown that mild or severe macro-steatosis increases the risk of EAD, primary non-function (PNF), vascular, and biliary problems in the early post-transplant period. It also affects tumor recurrence in recipients [18–20].

Omics data could address key issues in LT and describe the dynamic variations in molecules from a macroscopic perspective. Comprehensive and integrated multi-omics research could improve the understanding of molecular function and IR injury with mutual validations at all levels. Patients with initially poor function after LT using liver allografts in macro-steatosis status could be clearly distinguished by metabolomic features, and metabolomic data could contribute to monitoring long-term graft survival [21]. Our group conducted single-cell RNA sequence, demonstrated that Kupffer cells with high colony-stimulating factor 3 expression were proinflammatory and concentrated in transplanted steatotic livers, likely contributing to transplant damage [22]. Mass spectroscopy was used to parse metabolites differentiated between donations after circulatory and brain death. On this basis, a panel consisting of four metabolites was established to predict early graft function, and the metabolite classifier could stratify survival outcomes [23]. Proteomic and metabolomic examinations of hepatitis C virus-related liver illness showed enrichment of differentially expressed proteins linked to immunological, hepatoprotective, and fibrogenic processes, which can be used to predict early fibrosis [24]. Proteome profiling of mouse LT models was performed to determine the causes of cold and warm ischemic injury during transplantation. The mitochondrial membrane, endocytosis, and oxidative phosphorylation pathways were identified and focused. This study investigated the molecular functions that worsen steatotic donor LT damage and used molecule inhibitors to lessen transplant damage and increase LT options for patients with end-stage liver disease.

FABP4 is derived from metabolically active cells that regulate glucose and lipid homeostasis, inflammatory processes, and reactive oxygen species (ROS) production [25]. FABP4 deficiency is a promising treatment for insulin resistance, dyslipidemia, atherosclerosis, and other metabolic illnesses [26–28]. FABP4 was first identified in 2016 as a hypoxia-inducible gene, markedly induced by liver ischemia–reperfusion, and pharmacological inhibition of FABP4 could alleviate IRI in normal mice [29]. Our proteomic results revealed a difference in expression between normal and steatotic livers. Moreover, the difference existed in normal or steatotic livers compared to mice undergoing LT. Microarray results revealed that higher expression of FABP4 in the liver of donors is associated with poor survival prognosis and a higher incidence of EAD in recipients. The FABP4 inhibitor BMS309403 treats mice with acute liver injury and non-alcoholic steatohepatitis by inhibiting proinflammatory responses in isolated rat Kupffer cells [30]. Our results further revealed that FABP4 may represent a novel therapeutic target in steatotic LT, and FABP4 inhibitors may be used as therapeutic agents to manage IR injury in steatotic LT.

In a steatotic LT model, cAMP signaling cascade, aldosterone synthesis and secretion, and steroid hormone biosynthesis were annotated by integrated transcriptomic and metabolomic analyses after FABP4 inhibitor treatment. cAMP signaling was identified as a part of the cytoplasm-mitochondrion crosstalk that maintained mitochondrial homeostasis, regulates mitochondrial dynamics, and modulates cellular stress responses [31, 32]. Elevated cAMP signaling can inhibit mitochondrial fission, suspend unnecessary biogenesis, increased levels of dimerization and activity of ATP synthase and promote survival by sharing metabolites and boosting energy metabolism [33]. Manipulating mitochondrial cAMP signaling might modulate energy metabolism and control cell death, thereby managing mitochondrial dysfunctions. In addition, the roles of mitochondrial cAMP signaling and Epac1 (exchange protein directly activated by cAMP 1) in myocardial IRI have been verified. Inhibition of Epac1 could prevent hypoxia/reoxygenation-induced cardiomyocyte apoptosis by enhancing antioxidant capabilities, which is consistent with our multi-omics analysis [34]. Our results revealed that the relative expression of Hhip, Adrb2, Rac2, and PKA was significantly higher after the use of FABP4 inhibitors or siRNA, indicating the activation of the cAMP signaling pathway. We believed the adoption of FABP4 inhibitors might activate the cAMP signaling pathway, thereby responding to ischemia reperfusion injury or oxidative stresses to modulate a variety of mitochondrial behaviors including mitochondrial homeostasis and cell death.

ATP synthesis, intracellular Ca^2+^ modulation, ROS generation and clearance, and the management of apoptotic cell death are crucial tasks regulated by hepatocyte mitochondria, which are the cell’s powerhouses [35–37]. During steatotic donor LT, ROS may oxidize fat deposits to cause lipid peroxidation, which damages mitochondrial DNA to hamper the flow of electrons within the mitochondrial respiratory chain and produce more ROS [38, 39]. ROS-induced ROS production significantly increases the likelihood of mitochondrial autophagy [40]. TEM results depicted more autophagosomes in steatotic LT, and the mitochondrial structure was unclear. Rhodamine staining results suggest that FABP4 inhibitors could be used as therapeutic agents to manage IR injury in steatotic LT by regulating mitochondrial membrane homeostasis. The expression of mitochondria-related proteins exhibits a similar trend.

In conclusion, the mouse steatotic LT model demonstrated worse liver function than the normal LT model did. Proteomic results revealed that the mitochondrial membrane, endocytosis, and oxidative phosphorylation pathways were upregulated in the steatotic LT model. Differentially expressed proteins, which also interacted with fatty acid metabolism and synthesis, were screened in two of these four groups. FABP4 was discovered as a hypoxia-inducible protein that made recipients more susceptible to IR injury in steatotic donor LT. Adoption of an FABP4 inhibitor could protect the steatotic liver from IR injury during transplantation. Integrated omics analysis revealed that cAMP signaling pathway were enriched after using the FABP4 inhibitor while regulating mitochondrial membrane homeostasis in steatotic LT. The adoption of FABP4 inhibitors might activate the cAMP signaling pathway, thereby responding to ischemia reperfusion injury to modulate mitochondrial behaviors including mitochondrial homeostasis and cell death. We suggest that FABP4 is a novel therapeutic target and that FABP4 inhibitors could activate of cAMP signaling pathway thereby modulating mitochondrial membrane homeostasis, reducing oxidative stress injury in steatotic LT.

### Supplementary Information

Below is the link to the electronic supplementary material.

Supplementary file1 Establishment and validation of mouse sham model. A. Oil Red O staining of mouse sham model. B. H&E staining. C, D. TUNEL staining. (PDF 3577 KB)

Supplementary file2 Proteomic profile revealing the change during liver transplantation. A. KEGG pathway enrichment between NC SHAM and HF SHAM groups. B. Cellular component between NC SHAM and HF SHAM groups. C. Biological process between NC SHAM and HF SHAM groups. D. Molecular function between NC SHAM and HF SHAM groups. E. KEGG pathway enrichment between NC LT and HF LT groups. F. Cellular component between NC LT and HF LT groups. G. Biological process between NC LT and HF LT groups. H. Molecular function between NC LT and HF LT groups. (PDF 159 KB)

Supplementary file3 Figure 3. Transcriptomic profiles reveal the influence of FABP4 inhibitor. A. Genomic Circos plot, the outermost ring is the chromosome band, which displays the differential expression analysis results of different difference analyses from the outside to the inside. B. Reactome enrichment analysis between HF LT and HF BMS groups. C. DisGeNET enrichment analysis between HF LT and HF BMS groups. D. Disease ontology enrichment analysis between HF LT and HF BMS groups. (PDF 293 KB)

Supplementary file4 Table 1. Primary antibodies for Western blot assay. Table 2. Primers used for the target genes. (DOCX 56 KB)

Supplementary file5 (XLSX 1329 KB)

Supplementary file6 (XLSX 12544 KB)

## Data Availability

The data that support the findings of this study are available on request from the corresponding author. The data are not publicly available due to privacy or ethical restrictions.
