# Supplementary material for: Integrated multi-omic analysis identifies fatty acid binding protein 4 as a biomarker and therapeutic target of ischemia–reperfusion injury in steatotic liver transplantation
Source: Cell Mol Life Sci. 2024 Feb 10;81(1):83. doi: 10.1007/s00018-023-05110-1 (PMC10858962; doi:10.1007/s00018-023-05110-1)
Supplement: Supplementary file 1 — Supplementary file1 Establishment and validation of mouse sham model. A. Oil Red O staining of mouse sham model. B. H&E staining. C, D. TUNEL staining. (PDF 3577 KB) [file 18_2023_5110_MOESM1_ESM.pdf]

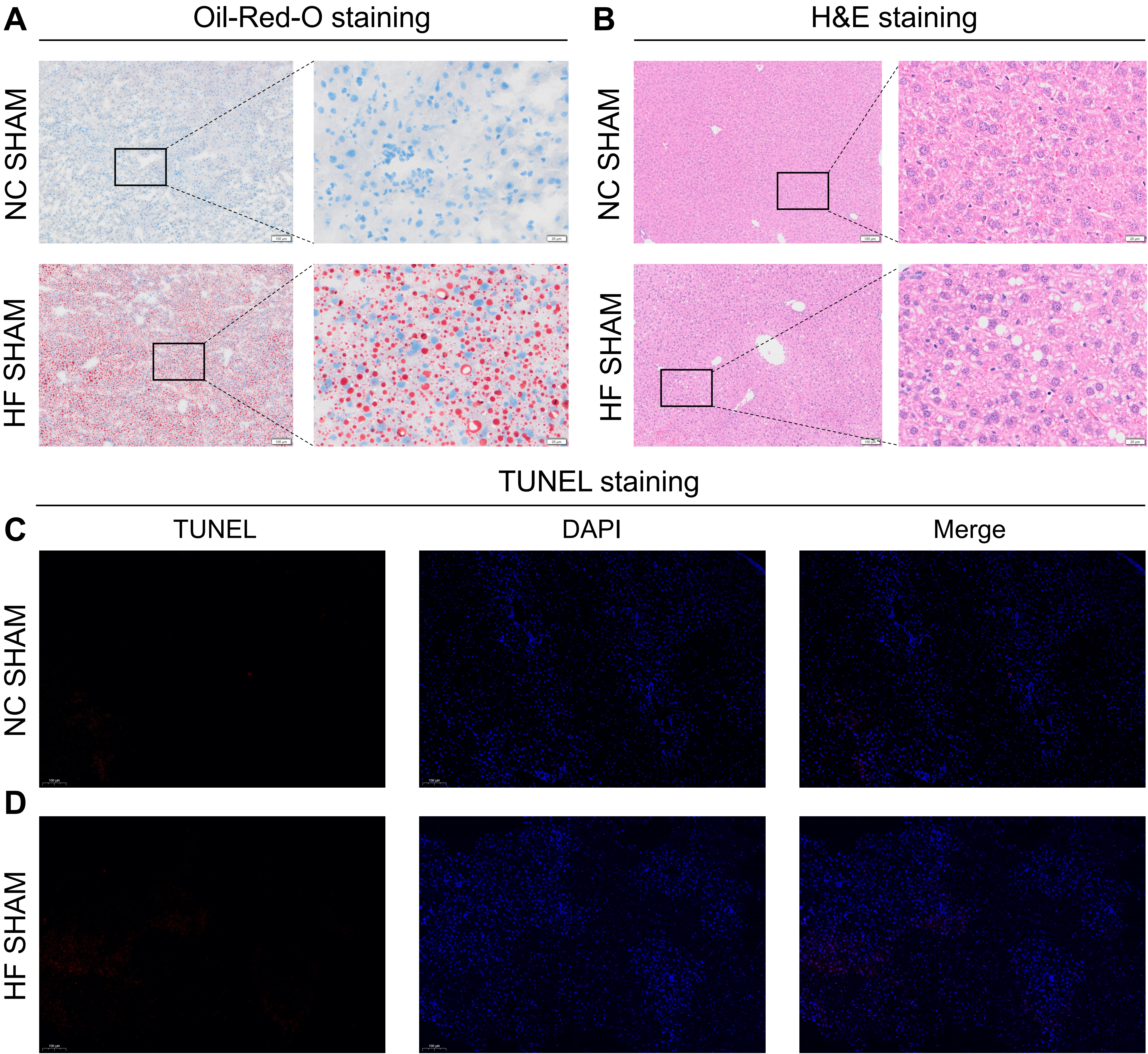
A
Oil-Red-O staining
B
H&E staining
NC SHAM
HF SHAM
TUNEL staining
C
TUNEL
DAPI
Merge
D
NC SHAM
HF SHAM
