# Supplementary figures and images for: Integrated multi-omic analysis identifies fatty acid binding protein 4 as a biomarker and therapeutic target of ischemia–reperfusion injury in steatotic liver transplantation
Source: Cell Mol Life Sci. 2024 Feb 10;81(1):83. doi: 10.1007/s00018-023-05110-1 (PMC10858962; doi:10.1007/s00018-023-05110-1)

A

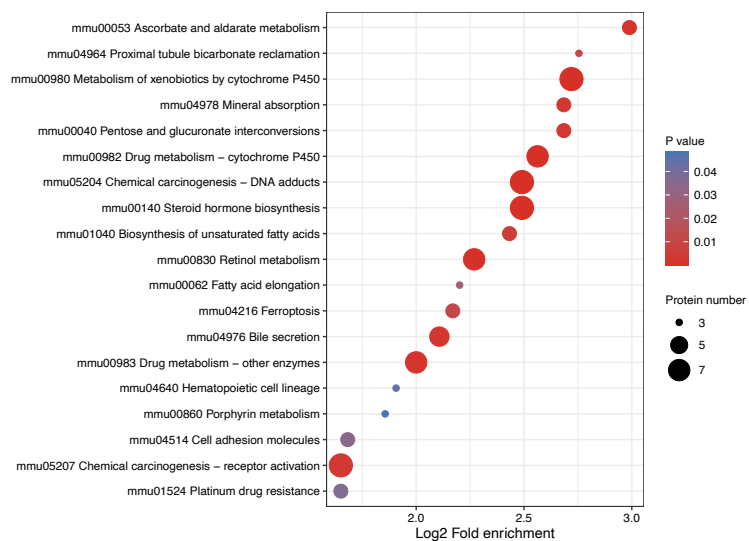

B

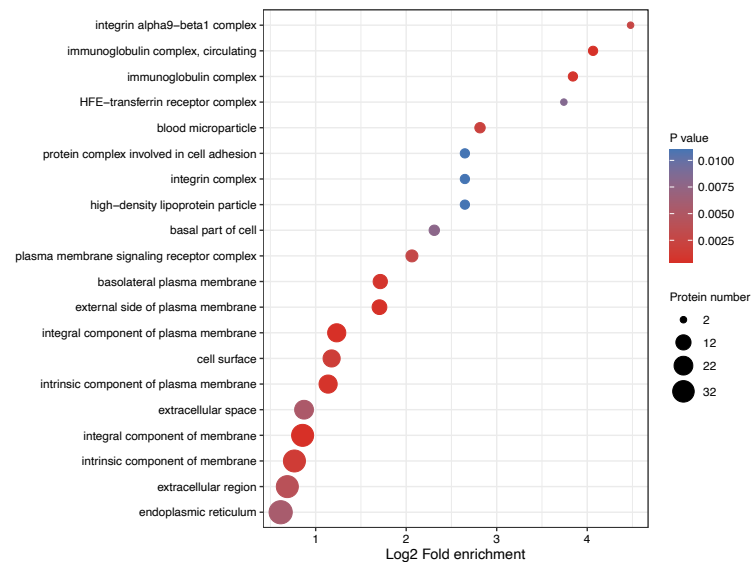

C

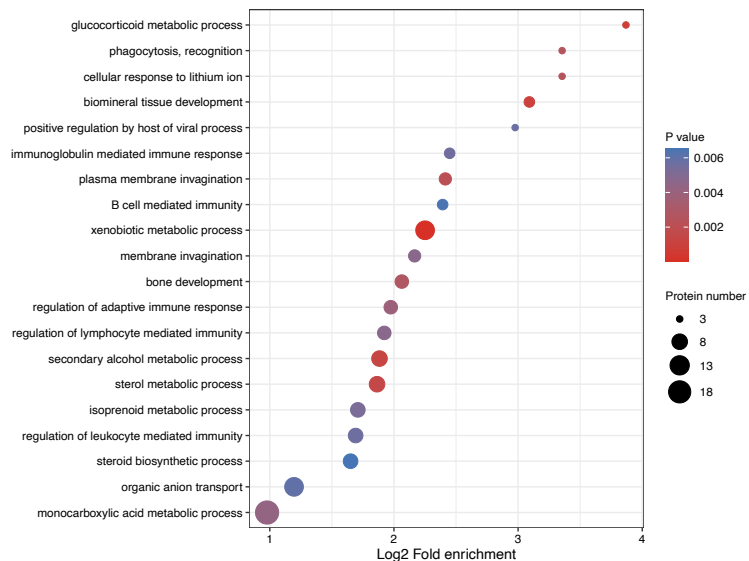

D

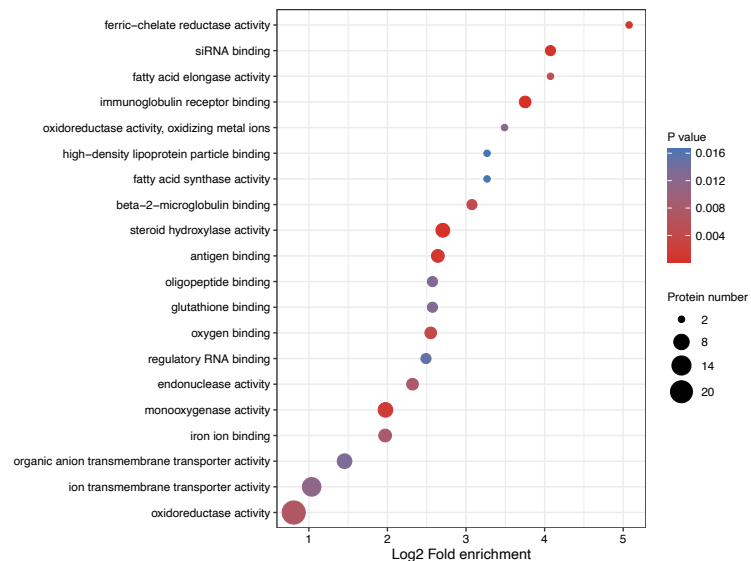

E

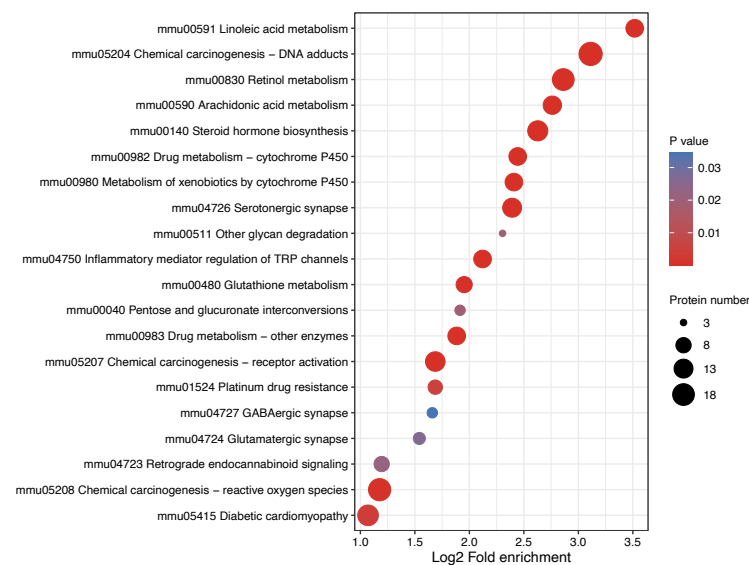

F

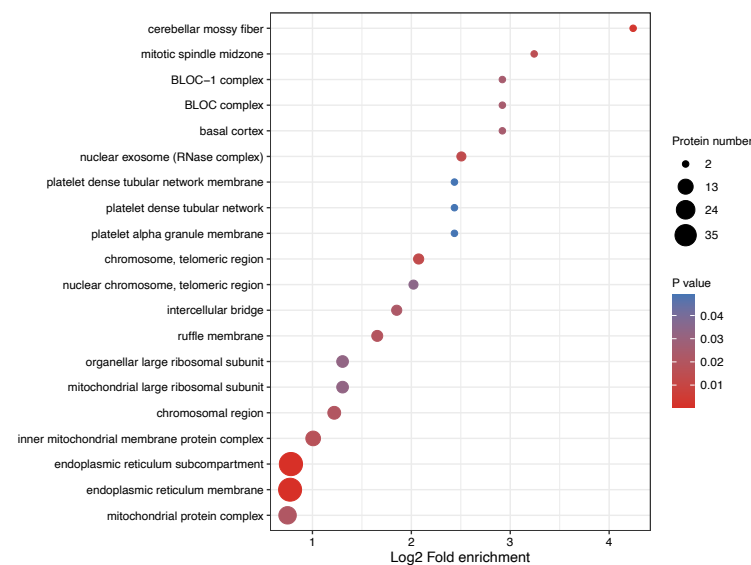

G

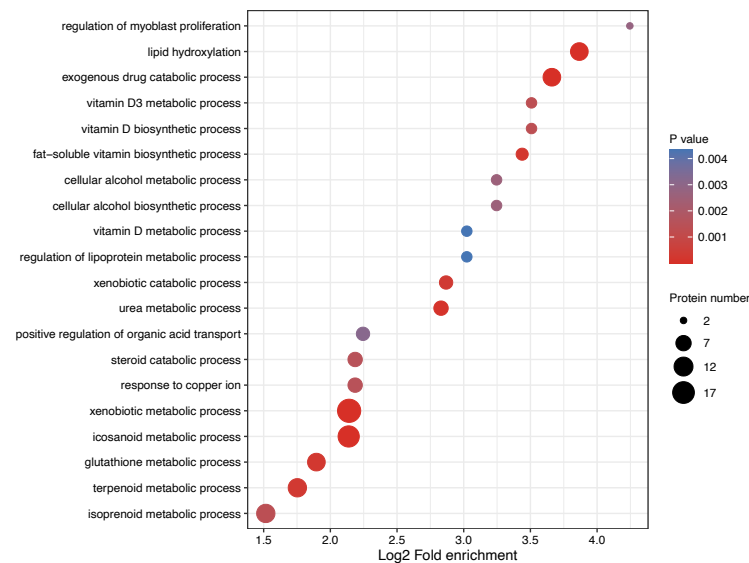

H

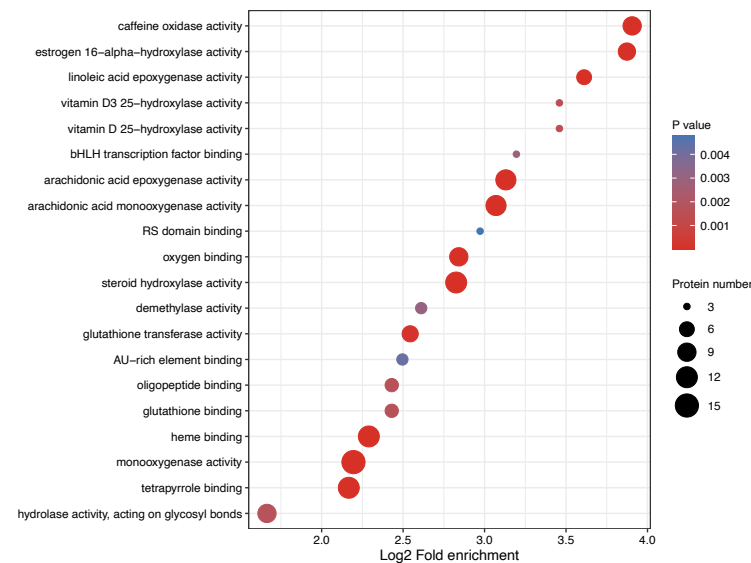

Supplement: Supplementary file 2 — Supplementary file2 Proteomic profile revealing the change during liver transplantation. A. KEGG pathway enrichment between NC SHAM and HF SHAM groups. B. Cellular component between NC SHAM and HF SHAM groups. C. Biological process between NC SHAM and HF SHAM groups. D. Molecular function between NC SHAM and HF SHAM groups. E. KEGG pathway enrichment between NC LT and HF LT groups. F. Cellular component between NC LT and HF LT groups. G. Biological process between NC LT and HF LT groups. H. Molecular function between NC LT and HF LT groups. (PDF 159 KB) [file 18_2023_5110_MOESM2_ESM.pdf]

A

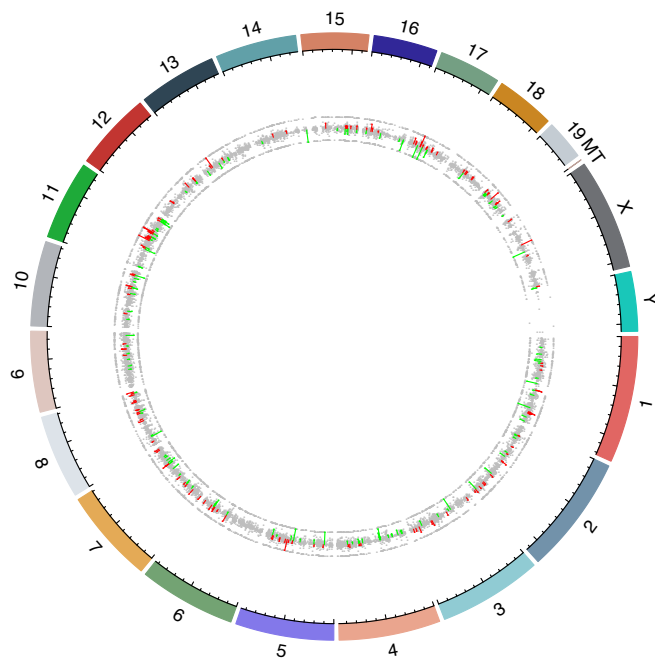

B

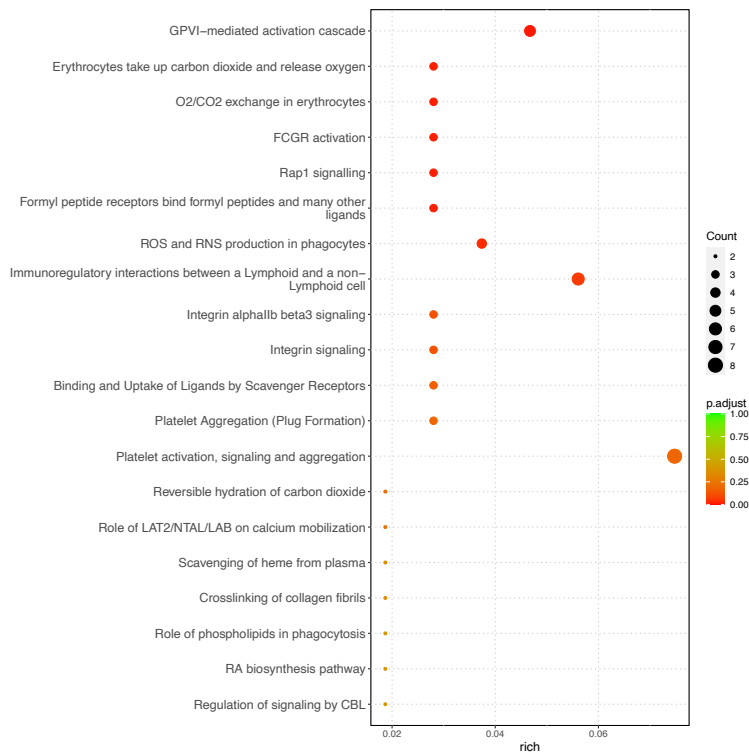

C

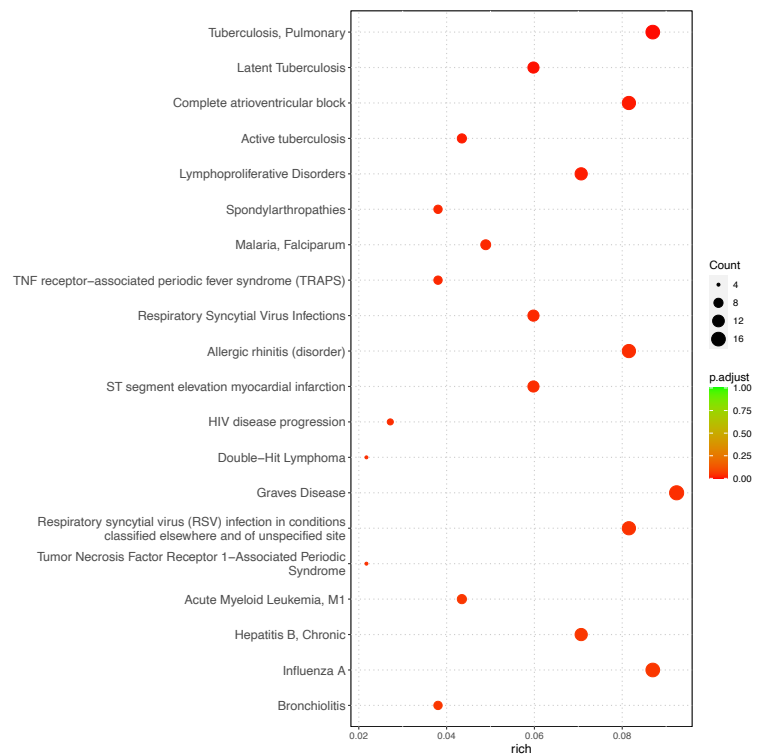

D

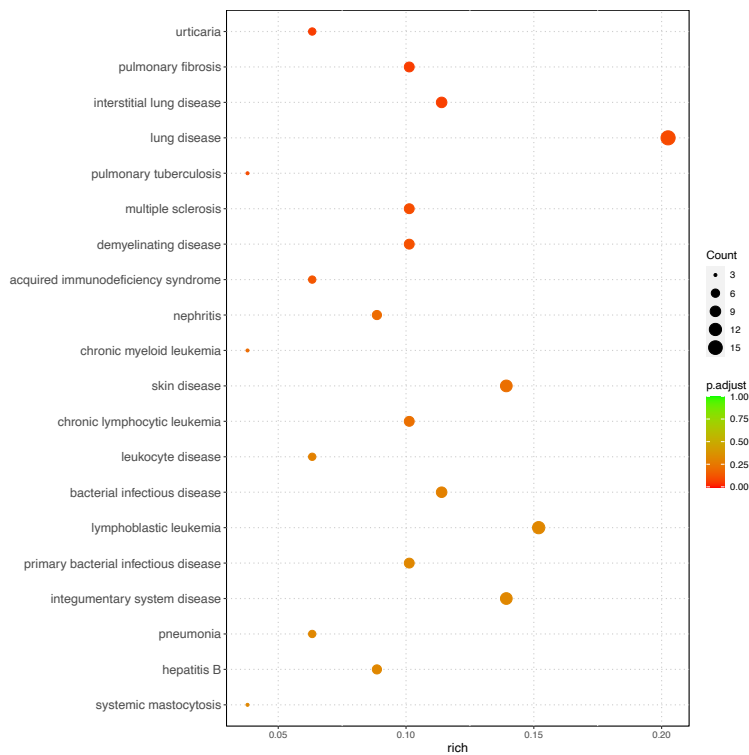

Supplement: Supplementary file 3 — Supplementary file3 Figure 3. Transcriptomic profiles reveal the influence of FABP4 inhibitor. A. Genomic Circos plot, the outermost ring is the chromosome band, which displays the differential expression analysis results of different difference analyses from the outside to the inside. B. Reactome enrichment analysis between HF LT and HF BMS groups. C. DisGeNET enrichment analysis between HF LT and HF BMS groups. D. Disease ontology enrichment analysis between HF LT and HF BMS groups. (PDF 293 KB) [file 18_2023_5110_MOESM3_ESM.pdf]
