# Supplementary material for: Integrated multi-omic analysis identifies fatty acid binding protein 4 as a biomarker and therapeutic target of ischemia–reperfusion injury in steatotic liver transplantation
Source: Cell Mol Life Sci. 2024 Feb 10;81(1):83. doi: 10.1007/s00018-023-05110-1 (PMC10858962; doi:10.1007/s00018-023-05110-1)
Supplement: Supplementary file 4 — Supplementary file4 Table 1. Primary antibodies for Western blot assay. Table 2. Primers used for the target genes. (DOCX 56 KB) [file 18_2023_5110_MOESM4_ESM.docx]

Supplementary table 1. Primary antibodies for western blot assay.

| Primary antibody | Co. Ltd. | Cat. # |
| --- | --- | --- |
| FABP4 | abcam | ab92501 |
| Bax | Cell Signaling Technology | #2772 |
| Cleaved PARP | Cell Signaling Technology | #9548 |
| PARP | Cell Signaling Technology | #9542 |
| Cleaved Caspase-3 | Cell Signaling Technology | #9664 |
| Caspase-3 | Cell Signaling Technology | #9662 |
| GAPDH | Cell Signaling Technology | #5174 |
| DRP1 | Cell Signaling Technology | #5391 |
| Mitofusin-1 | abcam | ab221661 |
| HHIP | Proteintech | 29466-1-AP |
| ADRB2 | Proteintech | 29864-1-AP |
| RAC2 | Proteintech | 60077-1-Ig |
| PRKACB | Proteintech | 55382-1-AP |

Supplementary table 2. Primers used for the target genes.

| Hhip | Forward | CAACCAGGAACGGTGGGCTATT |
| --- | --- | --- |
|  | Reverse | TCTGCGACTTCCAGAAACACCC |
| Adrb2 | Forward | GAGCGACTACAAACCGTCACCA |
|  | Reverse | TGGAAGTCCAGAACTCGCACCA |
| Rac2 | Forward | CTCAGCCAATGTGATGGTGGAC |
|  | Reverse | CGGACATTCTCATAGGAGGCTG |
| Adcy7 | Forward | GACGAGATGCTGTCAGCCATTG |
|  | Reverse | CACGCTCAAAGCCCTTCTCCAA |
| Gapdh | Forward | CATCACTGCCACCCAGAAGACTG |
|  | Reverse | ATGCCAGTGAGCTTCCCGTTCAG |

s
